# Internal Carotid Dissection as the Cause of Stroke in Childhood

**DOI:** 10.1155/2021/5568827

**Published:** 2021-06-28

**Authors:** Giulia Cinelli, Vitaliana Loizzo, Lisa Montanari, Ilaria Filareto, Elisa Caramaschi, Barbara Predieri, Lorenzo Iughetti

**Affiliations:** ^1^Post Graduate School of Pediatrics, Department of Medical and Surgical Sciences of the Mothers, Children and Adults, University of Modena and Reggio Emilia, Largo del Pozzo 71–41124, Modena, Italy; ^2^Pediatric Unit, Department of Medical and Surgical Sciences of the Mothers, Children and Adults, University of Modena and Reggio Emilia, Largo del Pozzo 71–41124, Modena, Italy

## Abstract

Internal carotid artery (ICA) dissection is a cause of stroke, but it is often underdiagnosed in children. ICAs' risk factors and pathogenic mechanisms are poorly understood, and the treatment is still empirical. We report the case of a previously healthy 9-year-old girl who presented with involuntary hypertonic closure of the right hand associated with transient difficulty for both fine movements of the right arm and speech. She had a history of minor cervical trauma occurring 20 days prior to our observation without other associated risk factors. Magnetic resonance imaging and magnetic resonance angiography showed ischemic lesions due to the left ICA dissection. Treatment with both acetylsalicylic acid and levetiracetam allowed recanalization of the ICA associated with the resolution of clinical signs. Our clinical case suggests that the ICA dissection must be suspected early whenever a child manifests mild neurologic deficits after a cervical trauma, especially if they are associated with headache and/or cervical pain. Moreover, the management of ICA dissection must be improved.

## 1. Introduction

Stroke is rare in childhood, but it involves severe morbidity and mortality. Congenital and acquired hearth diseases, vasculopathies, infections, genetic conditions, and metabolic disorders are the most common etiologic causes of strokes [1, 2] that can also occur after the dissection of cerebral vessels. Carotid artery dissection (CAD) accounts for 7.5–20% of ischemic strokes' causes [3, 4], and its recurrence in affected patients increases over time after the index event (5% at 1 month, 10% at 3 months, 12% at 6–12 months, and 15% at 60 months) [5]. Traumatic events, predisposing genetic conditions such as collagenopathies or states of hypercoagulability, and spontaneous dissections are risk factors for CAD development [6, 7]. Associated clinical signs are generally acute, mainly represented by visual disturbances, nausea, vomiting, headache, hemiparesis, and ataxia [8]. The magnetic resonance imaging (MRI) is firstly performed in order to detect the CAD and the brain damage, thereafter, during the follow-up [9, 10].

The American Heart Association/American Stroke Association reported guidelines for adults' stroke management [11] and provided indications for the clinical practice in children [12]. Published clinical trials recommended a therapeutic approach with antiplatelet and/or anticoagulant drugs [13, 14]. However, the usefulness of the antiplatelet therapy in patients with intracranial dissection is still controversial, and no consensus is still available on the duration of the anticoagulation therapy [1].

We report the case of a pediatric stroke due to CAD, characterized by neurological focal signs that occurred about 20 days after a minor cervical trauma.

## 2. Case Presentation

**Figure 1:**
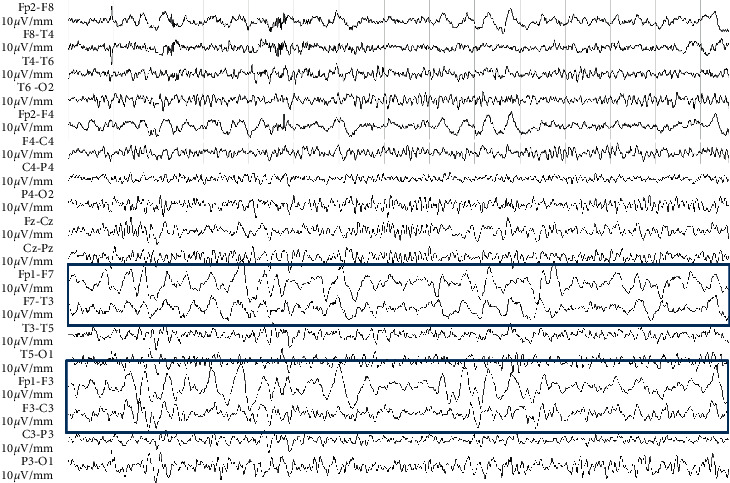
EEG pattern at the admission time—subcontinuous slow high-voltage activity focused on the left front-central regions.

**Figure 2:**
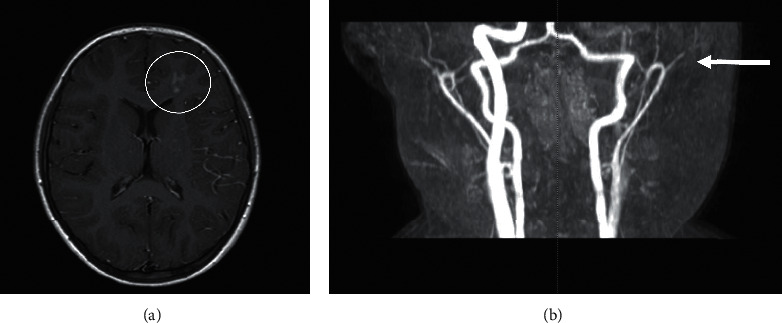
Brain neuroimaging results. (a) MRI: ischemic injury in the left frontoparietal region. (b) MRA: stenosis tightened by the left ICA and the thinning and irregularity of the left MCA.

A 9-year-old girl was admitted to our Pediatric Emergency Clinic because of brief involuntary hypertonic closure of her right hand, followed by transient difficulties in fine hand movements and in speech. These symptoms were also reported two weeks earlier, while other ones, such as headache, vomiting, and seizures, never appeared. The recent history was negative for severe traumas or injuries, while a mild cervical trauma occurred 20 days earlier due to the impact with the volleyball during the gym lesson. Her anamnesis was unremarkable, except for intermittent asthma and multiple allergen sensitization (dust mites, pollen, and canine grass), but no acute or chronic treatment was ongoing at the evaluation time. Family medical history was silent, except for one sister suffering from frequent vasovagal syncopal episodes, as documented by the tilt test. Specifically, connective tissue diseases or young onset strokes were not reported. At hospital admission, her body temperature, blood pressure, and other vital signs were in range according to age. Cardiopulmonary and abdominal physical examination was unremarkable. At the neurological examination, we found that tone, muscle trophism, strength, fine motility, and coordination were normal, as well as pupils were both same and normally reactive to the light. The evaluation of cranial nerves was normal. Romberg test, nose index test, and tightrope walker test were correctly performed. Reflexes were normal and symmetric. She did not refer sensitivity deficit. Laboratory test, including inflammatory markers and coagulation profile, resulted within the normal range (Table 1). In order to exclude epileptic seizures, the electroencephalogram (EEG) was performed, and a subcontinuous slow high-voltage activity focused on the left front-central regions was found (Figure 1). Brain MRI and magnetic resonance angiography (MRA) were performed for a better diagnostic definition, and hyperintense alterations of the frontoparietal signal, extended to the corpus callosum, were found in long-TR sequences (Figure 2(a)). These findings were consistent with a recent ischemic lesion. Another ischemic lesion in the late subacute phase was identified at the ipsilateral pale. There was no evidence of acute or chronic hemorrhage in both the subarachnoid space and the brain parenchyma. The MRA study showed both the tightened stenosis of the left internal carotid artery (ICA) and the thinning and irregularity of the left middle carotid artery (MCA), data that allowed us to diagnose the left ICA dissection (Figure 2(b)). The patient was then treated with acetylsalicylic acid (ASA, 100 mg/day) as antiplatelet therapy and with levetiracetam (up to 40 mg/kg/day) as antiepileptic therapy. The MRI was repeated 5 days after the therapy was started, and it showed an almost complete revascularization of both the carotid siphon and the left ICA, except for its lower cervical tract. The progressive revascularization of arteries was confirmed at subsequent MRI controls performed at 3, 12, and 24 months. Specifically, the MRI performed 24 months after the diagnosis showed the stability of the malacic-gliotic outcome of the frontal ischemic lesion and slightly reduced the caliber of the left ICA. During hospitalization, echocardiography, including the bubble study, was also performed to exclude malformations, mainly the patent foramen ovale and heart valve disorders. The autoimmune screening including antinucleous antibodies, neutrophil antigranulocyte antibodies, and rheumatoid factor was negative. Complement factors (C3 and C4) were in the normal range as well as homocysteine levels. The coagulation screening showed only lupus anticoagulant transient positivity (negative 12 weeks later). No mutation of the prothrombin gene, factor V Leiden, and von Willebrand was found. Finally, we excluded genetic connective tissue disorders such as Loeys–Dietz and Ehlers–Danlos syndromes. The patient was discharged after 15 days of hospitalization without clinical signs or symptoms. At the time of the submission of this case report, she is still in antiplatelet (ASA, 50 mg/day) and antiepileptic (levetiracetam, 45 mg/kg/day) treatment. After 24 months from diagnosis, her neurological examination is normal, and she complains only occasional headache. She is attending school with a good profit and socialization. Her follow-up is going to include clinical examination every 6 months, while MRI and echo color Doppler imaging of carotid vessels are going to be carried out every 12 months.

## 3. Discussion

Brain and neck CADs are major causes of cerebrovascular injuries in children [3, 4], and they may be spontaneous (commonly intracranial) or traumatic (commonly extracranial) [15]. The most common mechanism in traumatic CAD is direct blows to the neck/head or hyperextension [8, 9, 16]. Specifically, sudden acceleration/deceleration and rotation of the neck can stretch and compress the ICA causing dissection [6]. Different trauma types related to ischemic stroke cases are reported in Table 2 [4, 6, 7, 16–27], and it must be remembered that the trauma may be mild or even unnoticed [4, 5]. Sports, fights, and falls can cause CAD, but motor vehicle accidents play the most important role [8]. The annual incidence of spontaneous CAD was reported from 2.6 to 3.0/100,000 subjects [28]. Moreover, several other causes of spontaneous CAD were described, such as infection (i.e., varicella-zoster virus and pharyngeal infections), hereditary connective tissue disorders (i.e., Ehlers–Danlos syndrome), Moyamoya disease, arteriopathies, fibromuscular dysplasia, atherosclerosis, and cystic medial necrosis [4, 8, 29].

In our case, the patient reported only a mild cervical trauma that occurred about 20 days before symptoms' onset. No coagulation risk factors, connective tissue disorders, family history for coagulopathy, or dissection were found. The CAD can have different clinical presentations during childhood according to the cerebral site involved by the hypoperfusion. Neurologic signs could appear weeks after the trauma [19]. Moreover, hemiparesis, headache, aphasia, dysphasia, anopsia, and altered level of consciousness can frequently occur in affected patients [8]. In children, seizures were also observed, unlike in adults, while prodromal signs of upcoming acute ischemic stroke (just as transient ischemic attacks, amaurosis fugax, or local symptoms) were not frequently reported [12]. Our patient complained of mild, transient, and recurrent neurological symptoms. She had no seizures, and her neurological evaluation was normal.

Angiography is the gold standard for the CAD diagnosis; however, it is not often performed in children because arterial vascular access, general anesthesia, and ionizing radiation are needed to perform the exam. Nevertheless, MRI and MRA were described to be sensitive and specific as angiography. Specifically, MRI allows to detect acute ischemic stroke through a noninvasive diagnostic approach, giving a faster and safer diagnosis [9, 10]. In our case, despite the lack of typical seizures, we firstly performed the EEG, while MRI/MRA was performed thereafter, also according to the EEG result.

To date, efficacy and safety of the antithrombotic treatment (anticoagulants or antiplatelets) in patients with intracranial artery dissection are not yet assessed in randomized controlled trials, systematic reviews, and meta-analyses of observational data. Probably, mechanisms of cerebral ischemia in intracranial artery dissection are the same as those of cerebral ischemia in cervical artery dissection [29]. Markus et al. [14], in a trial including 250 adult patients (aged from 18 to 87 years) with cervical artery dissection, demonstrated that antiplatelet (mostly ASA) and anticoagulant drugs had the same efficacy for the prevention of stroke and death. ASA was also recommended for secondary prevention after transient ischemic attack (TIA) or ischemic stroke according to the trial data, showing a 13% reduction in the long-term risk of recurrent stroke. However, the risk of major stroke is very high only few days after TIA and minor ischemic stroke. Studies showed substantially great benefits of the early medical treatment in the acute phase [13]. In our patient, we decided to start with the antiplatelet therapy, assuming that thromboembolism was the cause of site stroke secondary to the CAD and taking into account the long time elapsed between the beginning of the first clinical signs and the access to our department. The clinical outcome was good with a fast and progressive arterial revascularization. Moreover, we started an antiepileptic therapy with levetiracetam because of its neuroprotective properties in both epileptic and nonepileptic conditions and its frequent use in the prevention of poststroke epilepsy in adults [30, 31].

## 4. Conclusion

CAD in childhood is a rare event, but it can be the consequence of a minor trauma. In suspected cases, the lesion can be safely detected through brain MRI/MRA. Despite treatment options which are not yet standardized in children, in our patient, both the antiplatelet and the neuroprotective therapies were useful to reach a final favorable outcome and no long-term complications. However, more studies on the therapeutic management of stroke and CAD in the pediatric population are needed. This clinical case allowed us to underline how the dissection of carotid and vertebral arteries is probably an underdiagnosed pathology in children. Despite the lack of treatment guidelines, according to our experience, we always suggest to include the ICA dissection in the differential diagnosis of a child presenting with mild neurologic deficits after a cervical trauma, especially if they are associated with headache and/or cervical pain.

## Figures and Tables

**Table 1:** Patient laboratory data at the admission time.

Coagulation function	(i) PT ratio: 1.02 (range: 0.84–1.20)
	(ii) PT INR: 1.02 (range: 0.84–1.25)
	(iii) APTT ratio: 1.03 (range: 0.80–1.25)
	(iv) Fibrinogen: 307 mg/dl (range: 200–400)
	(v) Homocysteine level: 7.28 *μ*mol/L (range: 5–12)
	(vi) Lupus anticoagulant research: positive
	(vii) Activated protein C resistance: negative
	(viii) Protein S: 0.77 U/ml (range: 0.53–1.09)
	(ix) Mutation of factor V Leiden: negative
	(x) Mutation of factor II: negative

Autoimmune investigations	(i) Complement factor
	(1) C3: 131 mg/dl (range: 80–190)
	(2) C4: 15 mg/dl (range; 15–57)
	(ii) Rheumatoid factor: 11 UI/L (normal value: <20)
	(iii) Antinucleous antibodies: negative
	(iv) Neutrophil antigranulocyte antibodies: negative
	(v) Anti-beta2glicoprotein IgG: 1.4 U/ml (positive value: >10)
	(vi) Anti-beta2glicoprotein IgM: 0.6 UI/ml (positive value: >10)
	(vii) Anticardiolipin IgG: 1,3 GPL-U/ml (positive value: >40; low positive range: 10–40)
	(viii) Anticardiolipin IgM: 1,3 MPL-UI/ml (positive value: >40; low positive range: 10–40)

**Table 2:** Pediatric case reports on CAD associated with minor head/cervical trauma.

Author/year	No. of patients	Age	Cause of injury	Symptoms	MRI or CT images
Borges et al. (2000) [16]	2	16 years	Intraoral trauma	Left hemiparesis, somnolence, confusion	Infarct/right MCA
		4 years		Left hemiparesis, aphasia	Infarct/left ICA

Bar et al. (2002) [17]	1	9 years	Intraoral trauma	Right hemiparesis, aphasia	Infarct/left ICA

Payton et al. (2004) [18]	1	11 years	Bumped head	Slurred speech and headache, tongue deviated to the right side and eyes deviated to the left, dysarthria, confusion	Infarct/left ICA

Agner and Weig (2005) [19]	2	4 months	Child abuse	Seizures, left gaze unsteady gait, tremors in the right arm	Infarct/left ICA

Pierrot et al. (2006) [20]	2	4.5 years	Fall	Confusion, left hemiplegia, central facial nerve palsy	Infarct/right ICA
		3.5 years		Asymptomatic	No infarct/right ICA

Jariwala et al. (2006) [21]	1	17 years	Motor vehicle accident	Increased confusion, lack of strength, sensation of the entire left upper extremity	Infarct/right ICA

Lin et al. (2007) [22]	1	7 years	Water slide injury	Headache, vomiting, neck pain, facial palsy, hemiplegia, and slurred speech	Infarct/right ICA

Levack et al. (2009) [23]	1	14 years	Shoulder belt	Right hemiplegia and aphasia	Infarct/right CCA

Moriarty et al. (2009) [24]	1	10 months	Intraoral trauma	Decreased level of consciousness, weakness	Infarct/left MCA

Agostini et al. (2013) [7]	2	7 years	Violent head hyperextension-rotation episodes	Irritability, right-sided weakness	Infarct/left MCA
		2 years	Vigorous somersaults	Headache and visual trouble	Infarct/left ICA

Nouh et al. (2015) [25]	1	4 years	Roller coaster ride	Left-sided weakness and left facial drop	Infarct/right MCA

Akbas et al. (2016) [26]	1	5 years	Water slide use	Slurring of speech, right-sided weakness	Infarct/left ICA

Bent et al. (2016) [27]	1	16 months	Intraoral trauma	Diminished left extremity movement, dysconjugate gaze, fluctuating mental status	Infarct/right ICA

Zant et al. (2017) [4]	1	4 years	Minor head trauma	Encephalopathy and left-sided hemiplegia	Infarct/right MCA and ICA

Cebeci et al. (2018) [6]	1	10 years	Minor shoulder trauma	Dysphasia, facial palsy	No infarct/right ICA

## Data Availability

All the data generated or analyzed during this study are included within this article and its supplementary information files. The data supporting this systematic review are from previously reported studies and datasets, which have been cited. The processed data are available in the supplementary file and from the corresponding author upon request.
